# Development of Unilateral Peri-Lead Edema Into Large Cystic Cavitation After Deep Brain Stimulation: A Case Report

**DOI:** 10.3389/fneur.2022.886188

**Published:** 2022-05-23

**Authors:** Yue Lu, Chang Qiu, Lei Chang, Bei Luo, Wenwen Dong, Wenbin Zhang, Hai-Hua Sun

**Affiliations:** ^1^Department of Functional Neurosurgery, Nanjing Brain Hospital Affiliated to Nanjing Medical University, Nanjing, China; ^2^Department of Neurology, Yancheng Hospital Affiliated Southeast University Medical College, Yancheng, China

**Keywords:** Parkinson's disease, deep brain stimulation, peri-lead edema, intraparenchymal cyst, subthalamic nucleus

## Abstract

**Background and Importance:**

Deep brain stimulation (DBS) has been approved to treat a variety of movement disorders, including Parkinson's disease (PD), essential tremor, and dystonia. Following the DBS surgery, some perioperative and even delayed complications due to intracranial and hardware-related events could occur, which may be life-threatening and require immediate remedial measures.

**Clinical Presentation:**

We report a case of an older woman with advanced PD who developed the unique complication of unilateral cyst formation at the tip of the DBS electrode after undergoing bilateral placement of subthalamic nucleus DBS. After a period of controlled motor symptoms, the patient showed new neurological deficits related to right peri-lead edema. However, the new neurological symptoms regressed quickly over several days with stereotactic implantation of a puncture needle to drain the cyst fluid without removing the affected lead.

**Conclusion:**

The occurrence of an intraparenchymal cyst following DBS surgery is a rare but life-threatening complication that could relate to edema around the electrodes or cerebrospinal fluid tracking. Stereotactic aspiration makes the intracranial cyst regress safely and effectively and ensures that the electrode is in the optimal position of the target nucleus to achieve an effective DBS surgery.

## Background and Importance

Deep brain stimulation (DBS) surgery is associated with a variety of complications ranging from intracranial adverse events to hardware malfunction (1, 2). Although DBS surgery is minimally invasive, it can cause immediate and severe complications like stroke or intracranial hemorrhage, occurring in ~1–2% of post-operative patients (3, 4). Furthermore, DBS requires chronic implantation of hardware leading to hardware-related complications such as infections (6.1%), migration or misplacement of leads (5.1%), lead fractures (5.0%), and skin erosion (1.3%) (5, 6). These complications lead to a decline in the quality of life in patients, and the previous benefits of DBS could be entirely lost (7). In this case report, we described a patient with Parkinson's disease (PD) who developed new neurological deficits 2–5 months following the DBS surgery due to peri-lead edema progressing into large cystic cavitation, revealed by imaging (8). We also reviewed the literature to discuss the potential etiologies and proposed some coping strategies for rare complications (9). We considered two possible explanations for intracranial cyst formation: (i) post-operative peri-electrode edema progressing into large cystic cavitation and (ii) cerebrospinal fluid (CSF) at the puncture point of the cerebral cortex flowing along the electrode toward the electrode tip (10). Given the rarity of this complication, there was no expert consensus on treatment. However, previous case reports reported conservative treatment with steroids, and cyst regression occurred along with clinical improvement (11). In addition, removing the affected lead or stereotactic cyst aspiration could be a potential supplementary treatment.

## Clinical Presentation

We presented a case of a 60-year-old right-hand dominant illiterate female patient who was diagnosed with PD approximately 12 years before undergoing DBS surgery. The patient had no history of chronic diseases like hypertension and diabetes, and no behavioral or cognitive complaints were reported. At first, the patient presented only with tremors on the right upper limb and a significant increase in muscle tone, which significantly improved with levodopa and dopamine agonists. At this time, the patient took half a tablet of Medopa (a tablet of Medopa including levodopa 200 mg and benserazide 50 mg,TID). However, the dosage and type of medicine were changed to one tablet of Madopa (QID) and pramipexole hydrochloride (0.25 mg, QID) as the PD progressed, but the duration of their effects shortened. Eventually, the drugs caused many side effects (such as dyskinesia, marked ON-OFF time, severe constipation, and insomnia) and severe motor fluctuations. Moreover, the body posture of the patient was abnormally accompanied by severe anxiety during the OFF-med, making walking extremely unstable and prone to falls. In order to resolve the above problem, the patient sought a surgical alternative. Therefore, preoperatively, the Unified Parkinson's Disease Rating Scale (UPDRS), Hamilton Anxiety Scale (HAMA), and Hamilton Depression Scale (HAMD) were performed by a neurologist with expertise in movement disorders to evaluate the patient depicting 60% improvement on part III (OFF-med:65 ON-med:26). The UPDRS score of the patient, combined with her neuropsychological evaluation (HAMA:3 HAMD:4) and routine preoperative biochemical examination, revealed no contraindications to DBS.

**Figure 1:**
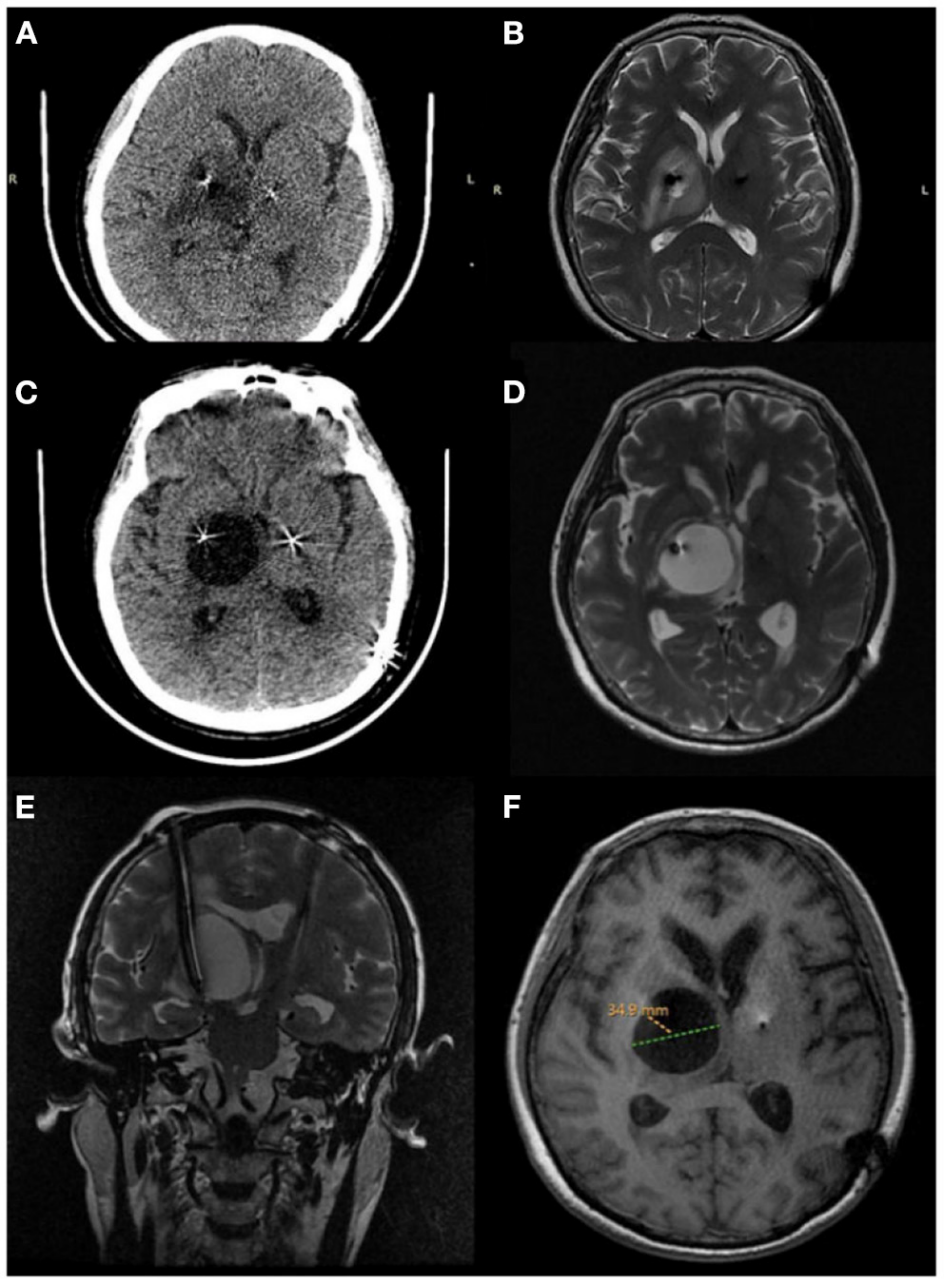
Peri-lead edema 2 months after deep brain stimulation surgery. **(A)** Axial CT, **(B)** axial MRI T2. Cystic cavitation around the lead 4 months after deep brain stimulation surgery. **(C)** Axial CT, **(D)** axial MRI T2. Five months after deep brain stimulation surgery. **(E)** Coronal T2 (MRI) showing a large cyst, **(F)** axial T1 (MRI) the cystic cavitation with a maximum diameter of 34.9 mm.

The patient underwent bilateral placement of DBS leads (L301, PINS, Beijing, China) targeting the subthalamic nucleus. The DBS operation was performed under the guidance of a surgical plan (pre-operative MRI fused with framed CT) and the monitoring of intraoperative electrophysiology. The electrodes were implanted into the predetermined targets on both sides in a single pass. After the operation, the vital signs of the patient were stable without intracranial hematoma or edema based on the immediate postoperative CT imaging. The leads were optimal, as confirmed by fusing the postoperative image with the preoperative surgical plan. The amount of medicine taken within 1 week after the operation was significantly reduced to only 1/4 tablet of Medopa (TID) compared with the previous one. Turning the stimulator on for initial programming 3 weeks after surgery resulted in tremor relief on the right upper limb and a steady walk even in OFF-med (UPDRS-III:41 improvement:36.9%) through routine programming settings (voltage: 1.5 V, pulse width: 60 μs, frequency: 130 Hz). However, during follow-up, the patient presented a continuous leftward tilt of the body with a static tremor of the right upper extremity without significant cognitive decline. The physical examination suggested: a general increase in muscle tone, especially on the right side. There was a right limb static tremor, and bilateral rotation movement was not coordinated. At the 2-month postoperative follow-up, an axial CT and 1.5 T magnetic resonance imaging (MRI) were performed. The images revealed hypodensities or high signals surrounding the right lead, extending from the subcortical region to the deep white matter near the lateral ventricles (Figures 1A,B). In the fourth and fifth months after DBS, CT and 1.5T MRI of the brain revealed a right cystic cavity with a maximum diameter of 34.9 mm compatible with CSF in all sequences surrounding both leads and their contacts (Figures 1C–F). In addition, MRI with gadolinium revealed significant right peri-lead edema with large non-enhancing cystic cavities along the leads extending from the subcortical region to the deep white matter near the lateral ventricles. At the same time, CT imaging revealed no evidence to support intracranial hemorrhage. The electrode tip was close to the cyst wall, located on the ventrolateral side. Notably, the patient did not demonstrate infection-related symptoms, including fever or leukocytosis, negative blood cultures, and healed surgical incisions. Furthermore, her erythrocyte sedimentation rate and C-reactive protein were within the normal range. However, the mood of the patient was more anxious than before the operation (HAMA:14 HAMD:15). Even when the stimulation was turned on, the patient exhibited a significant decline in her motor function. Moreover, turning off the device did not yield any improvement.

### Treatment

After obtaining the CT and MRI imaging features of this patient, the primary diagnosis we considered was a brain abscess. However, peri-lead edema and cavitation caused by infectious factors were ruled out based on the blood and the imaging results. Initially, this patient was given conservative treatment, such as steroid and antibiotic therapy and follow-up observation. However, the peri-lead edema did not abate and gradually developed into a large cyst over time (12).

**Figure 2:**
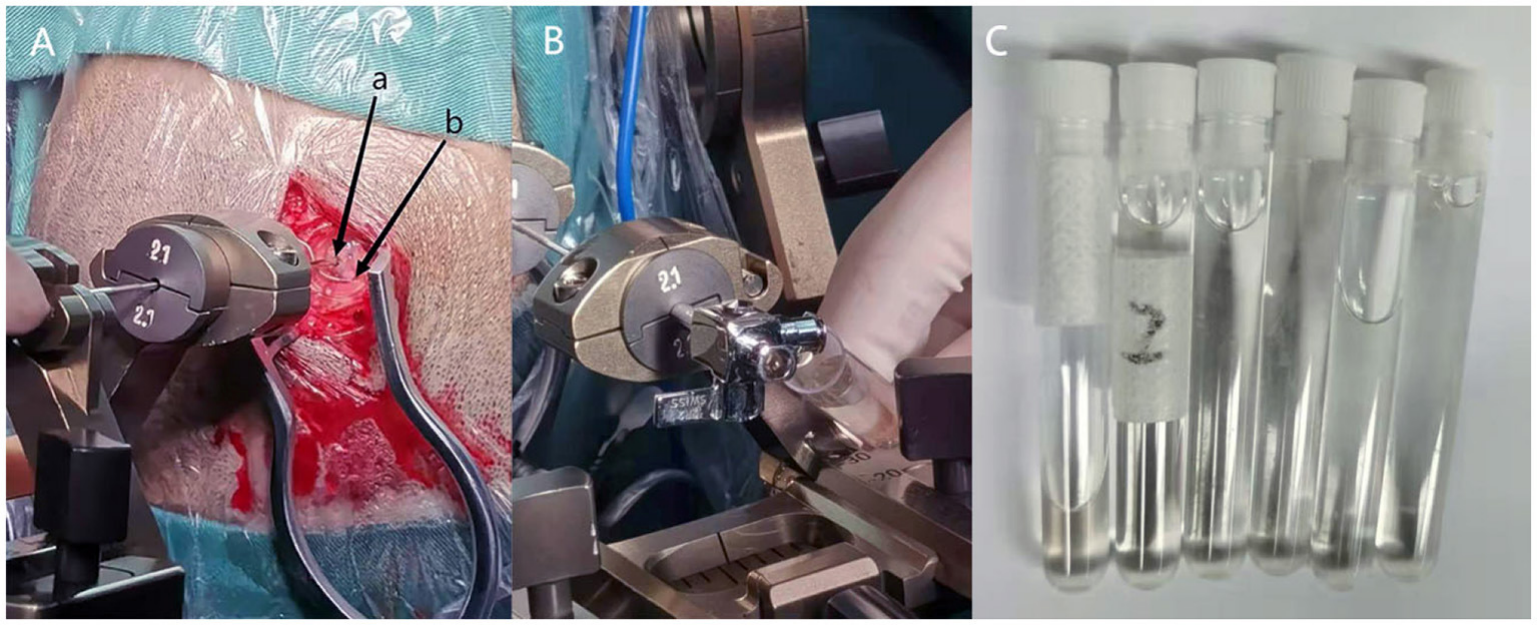
**(A)** Stereotactic cyst puncture is performed (a: Brain puncture needle hole, b: previous electrode hole). **(B)** The clear cyst fluid will flow out automatically after the brain puncture needle into the cyst. **(C)** About 25 ml clear cyst fluid drained through stereotactic cyst aspiration.

**Figure 3:**
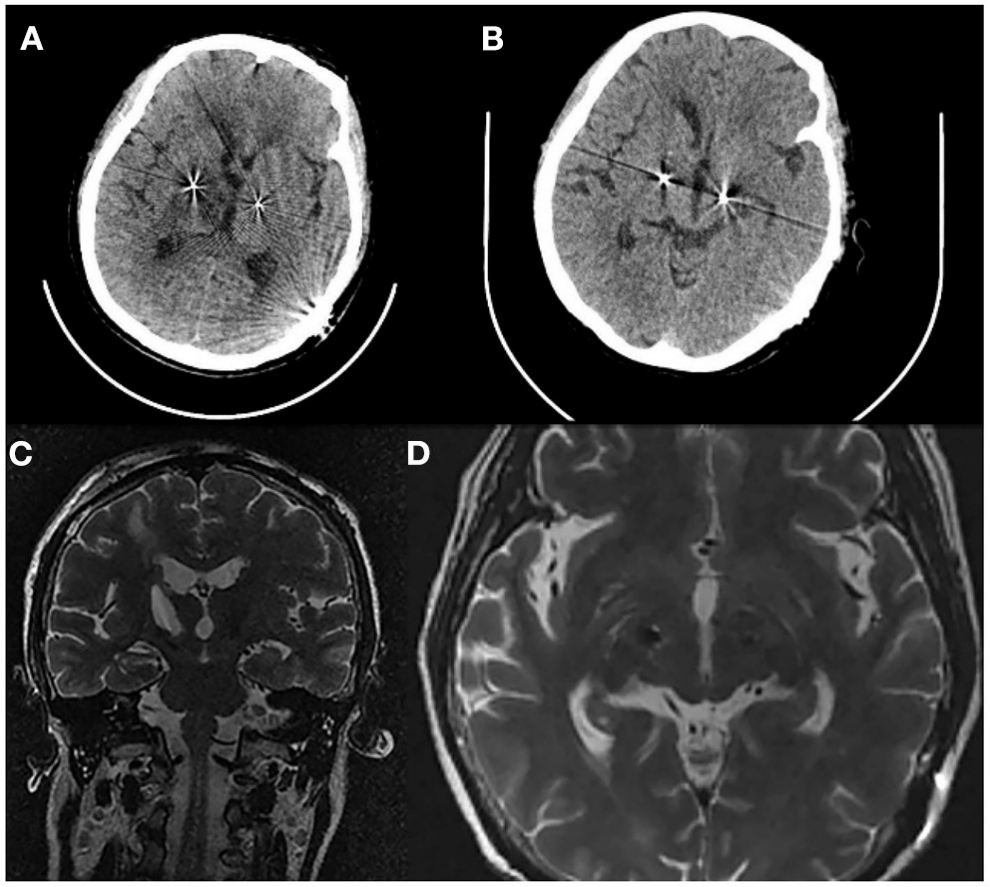
**(A)** Axial CT 1 day after cyst aspiration demonstrates the cyst that have almost disappeared. **(B)** Axial CT 6 days after cyst aspiration. **(C)** Coronal T2 (MRI) 6 days after cyst aspiration. **(D)** Axial T2 (MRI) 6 days after cyst aspiration without displacement of the right lead.

After discussing the risks, benefits, and alternatives with the family of the patient and obtaining consent, stereotactic aspiration of the cyst was implemented through a puncture needle without removing the affected DBS lead. Ultimately, about 25 mL of clear cyst fluid was drained (6) (Figure 2). In order to prevent the recurrence of the intracranial cyst, bioprotein glue was injected into the puncture hole to block the electrode path, as the cyst fluid no longer flowed out. Then, the cyst fluid was sent for biochemical examination, which indicated that the sac fluid was pale yellow, clear, positive for Pan's test, with a red cell count of 5 × 10∧6/L, a nucleated cell count of 36 × 10∧6/L, a glucose content of 2.59 mmol/L, the protein content of 3.70 g/L, and chlorine content of 116.6 mmol/L. Furthermore, the results of the culture depicted no colony growth. The patient was discharged with significant improvement in her symptoms and continued tremor resolution, with ON-stimulation following surgery (Figure 3).

### Outcome and Follow-Up

**Figure 4:**
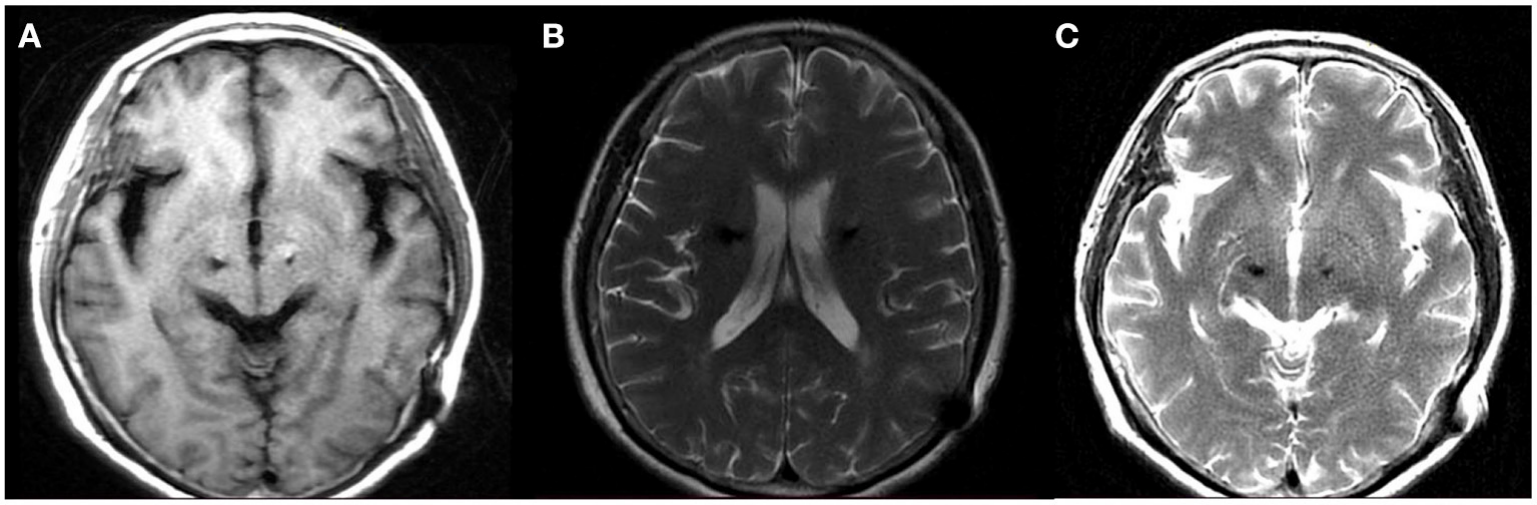
MRI following 5 month stereotactic aspiration demonstrated significant regression of the peri-lead edema and cystic cavitation. **(A)** Axial T1 **(B)** axial T2 **(C)** axial T2.

During the follow-up period after treatment, the patient was generally in good condition accompanied with 3/8 tablet of Medopa (TID). After a 5-month stereotactic aspiration, the patient was admitted for a 1.5T MRI in the OFF-stimulation state, demonstrating that peri-lead edema and cystic cavitation had significantly regressed without recurring (Figure 4). Furthermore, the patient underwent stimulation ON/OFF Unified Parkinson's Disease Rating Scale part III (UPDRS-III) testing in ON-med, and the result indicated an 18.6% improvement rate (OFF-stimulation:43 ON-stimulation:35) (13). Therefore, stereotactic cyst aspiration was the first measure that could be considered when conservative treatment methods were ineffective, and it ultimately ensured that the electrodes continued to deliver effective stimulation to improve motor symptoms. Under the combined treatment of the above medication protocol and programming (left:voltage: 2.0 V, pulse width: 60 μs, frequency: 130 Hz; right:voltage: 1.5 V, pulse width: 60 μs, frequency: 130 Hz), the patient's right upper limb tremor and muscle tension can be significantly reduced, and the patient can keep upright and walk independently.

## Discussion

This case reported a rare—but potentially catastrophic—adverse event associated with the DBS surgery: the development of peri-lead edema to a large cyst. It also demonstrated that, in the case of ineffective conservative treatment measures, stereotactic cyst aspiration without removing the affected electrode is effective and safe (14). Furthermore, after the cyst was stereotactically aspirated, the patient returned to upright posture without left-leaning within days. Our center has placed more than 700 leads since beginning the DBS surgery in 2010, and the core surgeon, equipment used, and target nucleus remain unchanged. However, this was the first time we had encountered an unexplained complication. Initially, we suspected it to be an intracranial abscess caused by an intracranial infection based on the imaging results. However, intracranial infections usually appear within the first few weeks following the DBS surgery and are accompanied by systemic symptoms, which are inconsistent with the state of the patient (15, 16). Furthermore, the negative results of the blood of the patient and post-operative cystic fluid culture proved that it was a cyst and not an abscess. Therefore, we hypothesized that the immune response of the patient to the implanted lead resulted in the cystic cavity depicted on imaging and explored the cause (17). If the above hypothesis were true, bilateral intracranial cysts would occur. However, the patient had only a single intracranial cyst on the right side and self-reported no underlying autoimmune disorder or history of severe allergies, overturning our previous assumption (18). Another possibility was that CSF from the subarachnoid space in the punctured area migrated down the affected lead and accumulated as a large cyst at the tip of the lead due to a backflow prevention mechanism, as previously described in another study (18). Importantly, the cyst gradually grew, causing a mass effect associated with neurological symptoms (19). In addition, the motor symptoms of the patient returned to the pre-operative baseline. They were not significantly alleviated whether stimulation was on or off, and some new problematic symptoms were even demonstrated.

We reviewed the relevant literature, and specific risk factors and potential pathological mechanisms of this complication remained unclear. Some centers reported that edema around the electrodes subsided using conservative treatment or removing the electrodes. However, in this case, we found that stereotactic cyst aspiration without removing the affected electrode should be given priority when conservative treatment measures were ineffective. We hope that this unique treatment experience provides valuable reference for other neuromodulation centers.

## Conclusion

As a rare but life-threatening complication, an intraparenchymal cyst after DBS lead placement would gradually disappear if the appropriate measures were taken. Therefore, steroid therapy and follow-up observation should be considered the first treatment choice. However, if these conservative treatment strategies do not work, stereotactic aspiration of the cyst without removing the affected DBS lead could be the best measure since it avoids the risk of secondary implantation of the electrode while ensuring that the electrode is within the optimal position of the target nucleus.

## Data Availability Statement

The original contributions presented in the study are included in the article/supplementary material, further inquiries can be directed to the corresponding authors.

## Ethics Statement

Written informed consent was obtained from the individual(s) for the publication of any potentially identifiable images or data included in this article.

## Author Contributions

All authors listed have made a substantial, direct, and intellectual contribution to the work and approved it for publication.

## Funding

This study was supported by the grant from subtopic of the 13th Five-Year National Key Research and Development Plan (Grant No. 2016YFC0105901NNZ) and Nanjing Health Science and Technology Development Special Fund Project (Grant No. ZKX20031).

## Conflict of Interest

The authors declare that the research was conducted in the absence of any commercial or financial relationships that could be construed as a potential conflict of interest.

## Publisher's Note

All claims expressed in this article are solely those of the authors and do not necessarily represent those of their affiliated organizations, or those of the publisher, the editors and the reviewers. Any product that may be evaluated in this article, or claim that may be made by its manufacturer, is not guaranteed or endorsed by the publisher.
